# Exploring strategies to connect formerly incarcerated individuals with community pharmacist-administered injectable naltrexone services

**DOI:** 10.3389/fpubh.2025.1514939

**Published:** 2025-07-18

**Authors:** Jason S. Chladek, Michelle A. Chui

**Affiliations:** ^1^NYU Grossman School of Medicine, New York, NY, United States; ^2^Division of Social and Administrative Sciences, University of Wisconsin-Madison School of Pharmacy, Madison, WI, United States; ^3^Sonderegger Research Center, University of Wisconsin-Madison School of Pharmacy, Madison, WI, United States

**Keywords:** community pharmacists, medication access, injectable naltrexone, opioid use disorder, formerly incarcerated individuals, community reentry, intervention, education

## Abstract

**Introduction:**

For formerly incarcerated individuals with opioid use disorder (OUD), the use of medications for opioid use disorder (MOUD) is critical, especially when these individuals transition out of correctional facilities and back into their communities. Unfortunately, few formerly incarcerated individuals use MOUD upon community reentry, often due to challenges with accessing treatment. As a result, this population remains at high risk of overdose and/or rearrest. In Wisconsin, community pharmacists are a promising resource for improving access and use of MOUD among formerly incarcerated individuals, specifically by administering injectable naltrexone. However, community pharmacists remain underutilized due to several barriers across the socioecological scale. Accordingly, this study used a participatory approach to explore strategies for addressing these barriers and connecting formerly incarcerated individuals to community pharmacist-administered injectable naltrexone.

**Methods:**

Five community pharmacists with experience administering injectable naltrexone and treating formerly incarcerated patients participated in three iterative semi-structured focus groups. The focus groups were conducted virtually, and Mural, an online collaborative whiteboard, was used to take notes on each focus group. Respectively, the goal of each focus group was to (1) discuss perceptions of existing barriers and prioritize barriers to be addressed based on perceived impact and feasibility, (2) identify and rank potential strategies for addressing the prioritized barriers based on perceived impact and feasibility, and (3) brainstorm strategy details/components and identify potential challenges related to the prioritized strategies. Focus groups were analyzed via deductive content analysis using a priori categories derived from the focus group goals.

**Results:**

In the first focus group, the participants prioritized two barriers to be addressed: lack of awareness of community pharmacist-administered injectable naltrexone services and lack of interagency collaboration among primary care clinics, community pharmacies, and correctional facilities. In the second focus group, the participants identified several strategies for addressing lack of awareness and/or lack of interagency collaboration, but prioritized pharmacist-led education targeted at correctional staff. Lastly, in the third focus group, the participants brainstormed several additional goals and topics for the educational strategy, including sharing existing resources, educating on required patient information, educating on providing patient information via prescriptions, establishing points of contact, emphasizing cost–benefits, and educating on the importance of insurance enrollment. Participants also identified potential challenges with the educational strategy, including inappropriate use of injectable naltrexone, time to implement educational sessions, and facilitating in-person meetings.

**Discussion:**

The findings provide a first step toward better leveraging community pharmacist-administered injectable naltrexone for formerly incarcerated individuals.

## 1 Introduction

Opioid use disorder (OUD) is defined as a problematic pattern of prescription or illicit opioid use, often leading to serious health and social consequences, including overdoses (1, 2). In Wisconsin, OUD has become a prevalent public health problem. From 1999 to 2019, there was a 900% increase in opioid overdose deaths (3). Notably, OUD is major problem among those impacted by the criminal legal system. From 2013 to 2019, the Wisconsin Department of Corrections reported 1,691 opioid-related hospitalizations among those admitted to probation and 754 opioid-related hospitalizations among those released from prison (4).

Medications for opioid use disorder (MOUD), which includes long-acting injectable naltrexone, are a critical component in treating OUD (5). Due to the high prevalence of OUD among those impacted by the criminal legal system, access to MOUD for this population is crucial. While continuation or initiation of MOUD within jails and prisons can still be improved, availability has expanded over the last decade (6–11). However, access to MOUD for individuals transitioning out of correctional facilities and back into their communities remains limited, partly due to inaccessible treatment locations and lack of care coordination (12). For example, in Wisconsin, less than half of jails provide community linkage to MOUD for individuals reentering the community (7).

For formerly incarcerated individuals, the use of MOUD is especially important during community reentry. The first few days after release from incarceration present the greatest risk of overdose, as tolerance to opioid is lost while in jail or prison (13). Formerly incarcerated individuals receiving MOUD are 85% less likely to die due to drug overdose in the first month after release and have a 32% lower risk of rearrest (14). Yet, because so many formerly incarcerated individuals do not have access to MOUD during this time, they remain at a 40-fold greater likelihood of overdose following release compared to the general population (15). Additionally, formerly incarcerated individuals account for up to 50% of overdose deaths in certain regions of the country (16, 17).

There is a clear need to improve access to MOUD for formerly incarcerated individuals during reentry. In Wisconsin, a potential resource that may help address this problem is community pharmacists. Since 2019, community pharmacists in Wisconsin have the authority to dispense and administer naltrexone injections, a treatment option that shows many benefits and is widely accepted among individuals impacted by the legal system (18, 19). To provide naltrexone injections, a community pharmacist must complete a training on non-vaccine injections. While individual patients still need to obtain a prescription, they can receive the injections directly within any pharmacy that dispenses them and has a trained pharmacist. Community pharmacists can also enter a collaborative practice agreement (CPA), allowing them to perform any patient care service delegated to them by a physician, though this is not mandatory for administering naltrexone injections (19, 20). Importantly, community pharmacies are considered one of the most accessible locations to receive care (19).

However, research shows that there are several barriers to connecting formerly incarcerated individuals with community pharmacist-administered injectable naltrexone as well. Examples include drug costs, siloed organizations, administrative constraints, lack of patient information, training requirements, lack of awareness of community pharmacist-provided services, and lack of patient insurance (20, 21). Overall, it is important that additional work be done to overcome these barriers. Accordingly, the goal of this study was to utilize a participatory approach to explore strategies for addressing the barriers and connecting formerly incarcerated individuals to community pharmacist-administered injectable naltrexone.

## 2 Methods and materials

### 2.1 Participants and sampling

Participants were recruited for three semi-structured focus groups between March 2024 and April 2024. Study participants included community pharmacists with experience administering naltrexone injections and treating formerly incarcerated patients. All participants were 18 years of age or older, able to speak and understand English, and residing in Wisconsin. The lead researcher (JC) had established connections to several community pharmacies across Wisconsin and leveraged these connections to identify and recruit participants. Initial recruitment was limited, so snowball sampling was used to identify and recruit additional participants who fit the inclusion criteria. The researcher ensured that participants were recruited from pharmacies in geographically diverse areas across Wisconsin. In total, five community pharmacists were recruited. None of the pharmacists worked for the same organization. The same five community pharmacists participated in all three focus groups. This study was deemed exempt by the University of Wisconsin-Madison Institutional Review Board.

### 2.2 Procedures

All potential participants were informed of the study and invited to participate via email. Once participants committed to the study, the lead researcher collected availability, and the focus groups were scheduled. An information sheet was then emailed to all participants. The information sheet was reviewed by the lead researcher on the call prior to the start of the first focus group, after which verbal consent to participate was obtained. The lead researcher emphasized that there was no obligation to participate, and participation was voluntary and could be stopped at any time. All focus groups were conducted via Zoom by the lead researcher, who had previous experience conducting semi-structured focus groups. Focus groups were audio recorded to help facilitate transcription and took 1.5–2 h each. After the focus groups, participants were sent a five-minute demographic survey. Participants were compensated with a $100 gift card for each focus group they participated in (up to $300 total).

The researcher conducted three semi-structured focus groups. The focus groups were iterative, with each focus group building off the previous one. Each focus group had specific goals, as outlined in Table 1. The goals aligned with the first three steps of a participatory design process. Participatory design has been shown to be beneficial in identifying strategies and designing interventions in complex work systems (22). For these goals, perceived impact was defined by which barriers could create the largest improvements if addressed and which strategies would be the most impactful in addressing the prioritized barriers. Perceived feasibility was defined by which barriers could be practically addressed and which strategies could be practically implemented within the existing system.

**Table 1:** Focus group goals and a priori categories for deductive content analysis.

Focus group	Goals	A priori categories
1	Discuss perceptions of existing barriers and prioritize barriers to be addressed based on perceived impact and feasibility	Perceptions of barriers Prioritized barriers
2	Identify and rank potential strategies for addressing the prioritized barriers based on perceived impact and feasibility	Strategy ideas Prioritized strategies
3	Brainstorm strategy details/components and identify potential challenges related to the prioritized strategies	Strategy details/components Potential challenges

*The table lists the goals of the three iterative focus groups, as well as the a priori categories used for coding during deductive content analysis. As shown, the a priori categories were developed from the focus group goals.

The research team internally developed semi-structured guides based on the goals of each focus group. For the first focus group, the participants were first presented with existing barriers related to community pharmacist-administered injectable naltrexone for formerly incarcerated individuals that were identified in a previous study (20, 21). The participants were first asked about their initial perceptions of the barriers, including if any came as a surprise. They were also asked to share any additional barriers that that were not presented by the researcher. Next, the participants were asked to prioritize which one or two barriers should be targeted first, thinking about perceived impact and feasibility.

The guide for the second focus group was developed after the conclusion of the first focus group. For the second focus group, the participants were asked to identify strategies that could address at least one of the prioritized barriers from the first focus group. The participants were then asked to select their top one or two strategies for addressing the prioritized barriers based on perceived impact and feasibility.

Finally, the guide for the third focus group was developed after the conclusion of the second focus group. For the third focus group, the participants were asked to identify and describe strategy details/components that should be included in the prioritized strategies. They were also asked to identify potential challenges related to the prioritized strategies.

Because the focus groups were iterative, the lead researcher utilized a digital whiteboard from Mural, an online collaboration tool, to take notes on the meetings (23). The digital whiteboard was shared in real time so that the participants could recall discussions from the previous focus groups, visually track the conversation, and make better connections between ideas. At the end of each focus group, participants were given the opportunity to ask any questions or share additional thoughts. All focus groups took place from April 2024 to May 2024.

### 2.3 Data coding and analysis

The focus groups were transcribed verbatim, de-identified and verified for accuracy. All participants were assigned an ID number. Transcripts were entered into NVivo, a qualitative data software package (released in March 2020) (24). As outlined by Elo and Kyngäs (25), two independent coders then performed deductive content analysis to place data into a priori categories. The a priori categories were based on the goals/questions from the focus groups and are outlined in Table 1. This analysis process was used, as participants occasionally discussed information that was relevant to goals/questions from a different focus group. For example, although the third focus group was focused on discussing potential challenges of the prioritized strategies, some participants mentioned potential challenges as they shared ideas during the second focus group. As a result, it was more effective to code all focus groups across the same categories.

After the coding process, the lead researcher summarized the data within each category. The research team met to discuss the summaries, as well address any ambiguities or issues related to coding. Finally, representative quotes were selected to support the results. Overall, the four-dimension criteria of qualitative research were used to guide the data coding and analysis process (26).

## 3 Results

In total, five community pharmacists participated in all three focus groups via Zoom. On average, the participants were 36 years old. Overall, 4 (80%) participants identified as White and 1 (20%) as Black/African American. None of the participants reported Hispanic or Latino origin or descent. Additionally, 4 (80%) of the participants identified as male, and 1 (20%) identified as female. All of the participants had a bachelor’s degree or higher.

Results from the focus group are described below and separated based on the focus group number. Overall, many of the participants expressed similar thoughts throughout the focus groups, especially regarding their perceptions of the existing barriers to community pharmacist-administered injectable naltrexone for formerly incarcerated individuals. Any variations or nuances between participants are discussed where applicable.

### 3.1 Focus group 1

Upon initial review, the participants stated that each of the barriers made sense and aligned with their perception of the current situation. One participant stated, “From my perspective, these all make sense. Especially knowing that not a lot of community pharmacies offer injectable naltrexone, at least to my knowledge,” (RPh1). The rest of the community pharmacists had similar reactions and, accordingly, none of them pointed to barriers that were particularly surprising. Only one barrier received minimal pushback, as one participant noted that stigma might not be a major barrier at every pharmacy, depending on whether or not the pharmacy has the ability to offer a private room for injections. If patients are aware that they can receive treatment privately, they may be less concerned with experiencing stigma. Additionally, none of the participants discussed additional barriers that were not presented by the researcher.

The participants also noted that many of the barriers overlapped. One pharmacist pointed out that, “All of them line up appropriately. Especially the collaboration with primary care and correctional facilities, which kind of goes hand in hand with them now knowing that community pharmacies are able to provide this service,” (Rh4). Another pharmacist noted that the inability of pharmacists to provide additional OUD services directly relates to the lack of available injection sites.

Based on perceived impact and feasibility, the pharmacists were asked to prioritize one or two of the barriers that they would address first. Unanimously, the participants selected lack of awareness of community pharmacist-administered injectable naltrexone services and/or lack of interagency collaboration among primary care clinics, community pharmacies, and correctional facilities. These barriers, as well as representative quotes, are shown in Table 2. As a result, these two barriers were used as the starting point for focus group 2.

**Table 2:** Prioritized barriers and representative quotes.

Barriers	Quotes
Lack of awareness	“Increasing awareness of services – from the patient all the way down to the person that is leading them out of the [correctional facility]. If they know that [community pharmacists] are involved in giving these injections, that’s huge. Increasing awareness, especially through increasing marketing, would be so much more impactful than connecting with [patients] by making a bunch of phone calls.” - RPh2 “Increasing awareness is [feasible]. It can happen through simple discussion with staff or with a provider. Like, ‘Hey, just to you know, we provide these services.’” - RPh1
Lack of interagency collaboration	“The collaboration. Because it is so important to make sure that the provider, the [reentry staff], and the pharmacist are on the same page.” - RPh1 “Community pharmacists have had a successful history of creating collaborations with physicians, with practitioners for other services. So, I think that would a feasible option here.” - Rh4

*The table lists the two barriers that were prioritized by the study participants during focus group 1. The barriers were prioritized based on discussions of perceived impact and feasibility. The table also highlights representative quotes from the focus group.

### 3.2 Focus group 2

Based on the results of focus group 1, the participants were instructed to consider the two prioritized barriers: (1) lack of awareness of community pharmacist-administered injectable naltrexone services and (2) lack of interagency collaboration between primary care clinics, community pharmacies, and correctional facilities. The participants were then asked to identify strategies for addressing these two barriers. Strategy ideas and representative quotes are outlined in Table 3.

**Table 3:** Strategy ideas and representative quotes.

Strategy ideas	Quotes
Development of a recovery clinic	“An opportunity could be, like, building out a recovery clinic where there are particular mental health providers, nurses, that can team up with community pharmacists that can dispense and provide the injection. I just think it might help to have, you know, streamlined services to specific clinics that might already have a rapport built up for opioid use disorder and have it be sent to those particular pharmacies that are providing those services.” - RPh5
Adding community pharmacists to existing online resources	“I think a good first step is to get in line with the Vivitrol website. I think it’s quite literally just vivitrol.com and you can find a provider. I think [providers] have to manually add themselves to that, so that’d be a really good starting point…getting [community pharmacists] as providers on that website would be a good first step.” - RPh1
Development of an informational website	“Maybe, you know, we do have a website for the case managers and those who are going to be helping connect the dots that states here are all the different, you know, pharmacies that are going to be giving long-acting injections in particular. You know, this is the insurance that they take…I think there could be a website specifically for opioid use disorder.” - RPh1
Development of an informational pamphlet	“We could have a little pamphlet that says, you know, it kind of goes through, you know, what opioids are. But on the front, there’s a little QR code…that brings them to [injectable naltrexone] near me or something along those lines. Or where I can find a pharmacy that carriers [injectable naltrexone] and accepts certain insurance.” - RhP4
Development of a central repository document	“I think there needs to be some type of central repository document. We already know that there are some places offering these services. But being able to see where these services are for, again, the staff that would help with reentry and ultimately that can get them connected to a pharmacy near that person’s home, that would be very helpful.” - RPh5
Pharmacist-led educational meetings with correctional staff	“Maybe setting up meetings with some of the [correctional staff] and just letting them know that this is something that we offer…I think it’s building that rapport and just opening the door and saying, ‘Hey, this is something that we are offering at the pharmacy.’” - RPh4 “Meetings are a great starting point. And I think education really needs to start [within corrections]. If you are looking at this specific patient population of how they are falling through the cracks and how they are winding up back behind bars, I think that is where it starts.” - RPh1 “I feel like we need to give education even further downstream. So, like, the prisons and facilities where those people who are giving the injections or the providers who did prescribe that injection in the prison can be able to then find the resources to connect them to the particular [community pharmacies] they could be. I almost feel like that’s where it should start at first.” - RPh5

*The table lists the strategy ideas that were shared by the study participants during focus group 2. Based on discussions of perceived impact and feasibility, the participants ranked pharmacist led educational meetings with correctional staff as the top strategy. The table also highlights representative quotes from the focus group.

Not only did several community pharmacists identify community pharmacist-led educational meetings with correctional staff as a potential strategy, but this strategy was almost immediately selected as the most important strategy by the participants. One participant stated, “Yeah, if there’s anything coming out of this, it’s education so that [correctional staff] understand that community pharmacies offer [injectable naltrexone] services and understand the steps to use them. That education needs to happen. It would be the best thing to come from this,” (RPh3). The rest of the participants agreed with this statement and added that educating correctional staff on the availability of community pharmacist-administered injectable naltrexone services could create a significant impact on connecting formerly incarcerated individuals to this treatment option. Additionally, the participants unanimously agreed that educational meetings would not only be a feasible option, but provide the best balance between perceived impact and feasibility.

The participants mentioned several other reasons that pharmacist-led educational meetings with correctional staff should be the strategy of choice. First, a few of the pharmacists stated that it is important to start at the source of the problem. Since formerly incarcerated individuals are reentering the community from correctional facilities, a strategy should be targeted at those who are involved in reentry at that point in time. Second, the participants explained that these meetings could accomplish several tasks. For example, the meetings could not only be used to increase awareness of community pharmacist-administered injectable naltrexone services, but could also help educate correctional staff on utilizing prescriptions and what patient information is required by community pharmacists, be used as an outlet to share existing resources, and allow pharmacists and correctional staff to establish points-of-contact. Importantly, the participants mentioned that these meetings could help address both of the prioritized barriers by increasing awareness and, in the long-term, increasing collaboration between community pharmacists and correctional staff.

In terms of the other potential strategies that were shared, a few community pharmacists noted that while some were good ideas, they may not be as impactful at improving access to community pharmacist-administered injectable naltrexone for formerly incarcerated individuals. For example, adding community pharmacists as providers to online resources may be helpful, but it would still require correctional staff and/or formerly incarcerated individuals to be aware of these resources and leverage the information on their own. Similarly, a few pharmacists noted that some of the strategy ideas would not be as feasible. Notably, while developing a recovery clinic could be very beneficial as a long-term goal, the participants mentioned that this would be difficult to implement as a first step. Accordingly, the participants ranked pharmacist-led educational meetings with correctional staff as their top strategy. This strategy was used as the starting point for focus group 3.

### 3.3 Focus group 3

Based on focus group 2, the participants selected pharmacist-led educational meetings with correctional staff as the top strategy. The participants agreed that the focus of the meetings should be educating correctional staff on the availability of community pharmacist-administered injectable naltrexone services. However, when asked to brainstorm and discuss additional details/components of the educational meetings, the participants fist highlighted other goals and topics for the educational meetings. These goals and topics, as well as representative quotes, are show in Table 4. The participants noted that adding these goals and topics could make the meetings more impactful without adding a significant amount of additional work.

**Table 4:** Additional goals and topics for educational meetings and representative quotes.

Goals and topics	Quotes
Sharing existing resources	“I’m a big fan of not reinventing the wheel. It would be helpful not to reinvent the wheel on everything. So, maybe some of these resources, like [vivitrol.com] could be shared during the meetings.” – RPh3
Educating on required patient information	“I think it’s important to educate on the things that are required at the pharmacy end. I think it needs to be known really by anyone who is involved with injections or reentry. Things like a diagnosis code, history of using [injectable naltrexone], date of last injection, if they have tried oral naltrexone…” - RPh4
Educating on utilizing prescriptions to provide patient information	“In an ideal world, we would love to be connected with EHR. But I even think on the prescription itself, there’s an area that says ‘Pharmacy Notes’ that you can actually input criteria, like, you know, ‘Yes, they are a candidate, this is the last time they took the drug, they have taken this medication before…’ I think that would be helpful for the [correctional staff] to know, so [the pharmacist] can have some information if they are not connected to the HER.” - RPh5
Establishing points-of-contact	“Sometimes you are trying to call and, you know, schedule appointments…or if [a formerly incarcerated individual] missed their appointment to try and call and get them back in for, you know, a reschedule…quite often their phone number changes, or their voicemail box has not been set up. Or you do not even have an actual address on file because they are kind of in that transitional stage where they are moving around and kind of getting reestablished. So, using [the meetings] to establish points-of-contact or contact info for social workers or case management can be huge.” - RPh1
Emphasizing cost–benefit	“Make sure you mention any kind of monetary incentive for them because it’s expensive to have somebody in jail and go back to jail. Injectable naltrexone is also expensive, but I could only assume that having them on monthly injection as opposed to having them in jail for another month at minimal…there’s a benefit of savings right there.” -RPh4
Educating on importance of enrolling individuals in insurance	“Maybe just insurance considerations. If we are talking about someone transitioning from a correctional facility to home or wherever, insurance factors into that. So, maybe including insurance considerations. And making sure that, like, the reentry staff knows they need to help these individuals kind of access insurance first before anything.” - RPh1

*The table lists additional goals and topics for the educational meetings that were shared by the study participants during focus group 3. Participants were asked to brainstorm strategy details and/or components and identified these goals and topics that could be included in the educational meetings, in addition to educating on the availability of community pharmacist-administered injectable naltrexone services. The table also highlights representative quotes from the focus group.

The participants also talked about how the educational meetings should be delivered. Overall, two main considerations emerged. First, the pharmacists all agreed that the meetings should be held in-person. One participant said, “I think in-person meetings are always going to be a lot easier and more people are able to digest more information,” (RPh3). Another stated, “I vote in-person. I think you can build more relationships that way and you can, you know, answer questions that might come up a little bit easier if you are in person. Things you might not have thought of when you were developing a web module or handout,” (RPh4). A third added, “Yeah, I second or third in-person. For me, I think it’s like, you know, building those relationships with people and kind of being able to express how emotionally invested you are as opposed to trying to imitate that via a webinar…I think having somebody in-person that can really say, ‘I’ve seen this change people’s lives.’ Simple as that,” (RPh1).

In addition to pushing for in-person meetings, the participants agreed that the educational meetings should be led by pharmacists who have experience providing naltrexone injections and working with formerly incarcerated patients. “I think it’s definitely easier for a pharmacist that’s already established [these services] to kind of take the lead on this,” stated one pharmacist (RPh1). Another echoed this thought and added, “And if you have somebody from a community pharmacy that is already offering this, you automatically make that connection. So, a really good strength of having [pharmacists with experience] lead is that you are creating those connections right away for those [correctional] facilities,” (RPh4).

Additionally, while the participants agreed that correctional staff (including correctional providers and reentry coordinators) should be the main focus of the educational meetings, there were a few other stakeholders that the participants thought could either improve the meetings or benefit from the information being shared. One participant said that drug representatives could support the pharmacists in educating correctional staff. “One [stakeholder] that comes to mind is drug reps…They have the time and they are getting paid, and they can help with the educational piece,” (RPh1). Another participant added, “I think what we are missing here is not involving social work or case management in the discussion. They really help bridge, so I would actually add having them involved in the discussion when you are having these in-person meetings,” (RPh5). Lastly, one pharmacist said that it would be beneficial to involve governmental officials. They said, “I would say include someone as high up in the government for the state as you can, too. Because if you can get, like, governor’s office on board or whoever the state overseer for correctional facilities is, like, and we make it a state priority, I think you’ll get a lot more buy-in from the facilities themselves,” (RPh4).

When asked about potential challenges related to the educational strategy, three main ideas were shared. One participant stated, “Providing education solely on naltrexone, on the injectable form, could lead – especially if they do not have a healthcare background – it could lead to some institutions just automatically jumping to injectable in patients that it’s not ideal for, which is a huge risk to that person and could lead to some really poor outcomes for those folks,” (RPh1). Ultimately, the participant was concerned that injectable naltrexone may be used in cases where other MOUD treatment options are more beneficial. However, the participant also acknowledged that the main goal of the educational meetings would be to increase awareness of available treatment options, specifically those provided at community pharmacies. They said that educating on this option could still help improve access for formerly incarcerated individuals needing injectable naltrexone and some progress is better than doing nothing – a thought echoed by the other participants. Additionally, one participant said that time might be a challenge or barrier. “I think a second thing is that if we focus on individual education or, like, institution to institution, it’s going to be very time consuming, even if we have the partnerships and everything like that,” (RPh1). Lastly, some of the pharmacists expressed concerns about who would be able to attend in-person meetings and whether or not those in rural areas would be excluded. “And obviously there’s going to be a lot of places in the rural settings that they are not able to meet in person, so showing that you are, like, fully invested in this, that would be very helpful,” (RPh5).

## 4 Discussion

Throughout the study, participants were given the opportunity to discuss and prioritize barriers related to community pharmacist-administered injectable naltrexone for formerly incarcerated individuals, as well as identify a strategy for addressing these barriers. Across all three focus groups, there was high level of agreement among the participants. The participants had similar perceptions of the existing barriers, agreeing that the barriers were not surprising and aligned with their own knowledge and experiences.

Overall, the participants not only unanimously agreed on the barriers that should be addressed first (lack of awareness and lack of interagency collaboration), but that a strategy to address these barriers should include education targeted upstream with correctional staff. This is important for three reasons. First, we know that the first several days after community reentry present the greatest risk for formerly incarcerated individuals with OUD. As a result, educating correctional staff can help ensure that formerly incarcerated individuals are connected to accessible community-based treatment as soon as they leave the correctional facilities. For example, it would also be beneficial to implement a strategy that increases the number of community pharmacists providing injectable naltrexone or other OUD services. However, without awareness of these services among correctional staff and/or collaboration between correctional facilities and community pharmacies, formerly incarcerated individuals may still be left to find and access these services on their own. Second, previous research has shown the education can be a successful strategy for facilitating the implementation of healthcare practices in correctional settings (27). In Wisconsin, the Department of Corrections (DOC) previously created trainings to educate staff about the three approved MOUD options (4). Ultimately, the educational strategy aligns with previous findings and other opioid-related efforts already implemented in the state. Third, pharmacist-led educational meetings could help address both lack of awareness and lack of interagency collaboration, which may make this strategy especially impactful.

On top of educating correctional staff on the availability of community pharmacist-administered injectable naltrexone, the participants identified other topics that could be discussed during the meetings. Several of these topics could help address other existing barriers identified in the literature (20, 21). For example, the participants stated that during the educational meetings, the pharmacists leading the meetings could educate on the importance of enrolling individuals in insurance before they are released. This can push reentry staff to make enrollment a priority, helping to increase insurance access for formerly incarcerated individuals before they are back in the community. Overall, these additional topics could help the educational meetings become a multipurpose strategy. The participants also identified several other stakeholders that could benefit from the participating in the educational meetings in addition to correctional staff. These included drug representatives, social workers and/or case managers, and governmental officials. Including these professional groups in the educational meetings could not only incorporate other perspectives, but further improve awareness of community pharmacist-administered services and foster even more collaborative relationships.

One stakeholder that was not mentioned was community health workers. Community health workers are individuals from the community who form relationships with individual patients and assist them in accessing health care and health-related resources (28). Importantly, community health workers can help patients overcome barriers related to the social determinants of health. Previous work has shown the benefits of collaborations between community health workers and pharmacists in improving patient outcomes (28, 29). As a result, including community health workers in the educational meetings could also prove to be beneficial, especially considering many of the obstacles that formerly incarcerated individuals face in accessing care. Furthermore, while the study participants agreed that the educational meetings should be led by pharmacists with experience administering injectable naltrexone and treating formerly incarcerated individuals, it may be beneficial to include community pharmacists who do not have these experiences. This could offer these pharmacists the chance to learn more about integrating injectable naltrexone services into their practice, especially considering the number of community pharmacies that provide injectable naltrexone throughout Wisconsin is limited (30). It could also push them to receive the training for administering non-vaccine injections and help them understand the impact they can make by connecting with and treating formerly incarcerated individuals. Importantly, previous research also shows that education can increase the implementation of new services within healthcare settings, including community pharmacies (31, 32).

As demonstrated by the third focus group, the educational strategy is not without potential challenges. However, there are ways to potentially eliminate or at least mitigate some of these concerns. For example, one participant expressed concerns about only educating on injectable naltrexone, stating that correctional staff may not be aware of other, possibly more appropriate MOUD options. To address this problem, the pharmacists leading these meetings could educate on which patients benefit the most from injectable naltrexone and how to identify these patients. They could also briefly discuss the other forms of MOUD and highlight some resources that provide guidance on accessing these options if necessary. As mentioned, some correctional staff members may already be aware of the MOUD options due to the trainings created by the Wisconsin DOC (4). Additionally, the participants said that educational meetings could be time consuming, and some expressed concerns that correctional staff in rural areas may be excluded. These challenges could be mitigated by coordinating meetings that involve correctional staff across a certain region of Wisconsin. By scheduling these meetings in advance and utilizing central locations, those residing in rural areas may have an easier time attending. At minimum, recordings could be sent to those who are unable to attend in-person meetings.

This study creates several directions for future work. Next steps could include the development and implementation of the educational meetings. Additional research could then be done to assess the efficacy of the educational meetings on increasing correctional staff knowledge of community pharmacist-administered injectable naltrexone services, fostering collaborations between correctional facilities and community pharmacies, and facilitating the use of community pharmacist-administered injectable naltrexone by formerly incarcerated individuals upon reentry. Demonstrating efficacy of the educational meetings could support the scale-out of the strategy to areas outside of Wisconsin with similar legal landscapes and work systems.

### 4.1 Limitations

There are certain study limitations that should be noted. For starters, because the study used convenience and snowball sampling to recruit community pharmacists, it is possible that bias was introduced. Additionally, the community pharmacists included in this study were from several counties in Wisconsin, including urban and rural areas. However, since pharmacists from every area could not be included, it is possible that the results do not represent the opinions and ideas of all pharmacists across Wisconsin. The results may also not be generalizable to areas outside of Wisconsin, especially those with different scope of practice laws and/or work systems. The study was also limited to community pharmacists who had experience administering injectable naltrexone to formerly incarcerated individuals. This not only limited the sample size, but may have influenced their decisions regarding the barriers and potential strategies. Ultimately, the inclusion criteria and nature of the selected strategy likely limit next steps to areas that have existing community pharmacist-administered injectable naltrexone services. It is also likely that the pharmacists who chose to participate in this study were more open to collaboration, potentially influencing their perceptions and limiting generalizability. Finally, the participants were predominantly male, white, and did not identify as Hispanic or Latino, resulting in a homogenous sample. Despite these limitations, this study was intended to be exploratory in nature, and additional work can help ensure the transferability of results.

## 5 Conclusion

It is crucial that formerly incarcerated individuals have access to MOUD upon release from correctional facilities. However, many of these individuals face barriers in accessing MOUD upon community reentry, incurring a high risk of overdose and/or rearrest. Community pharmacists are a promising resource for improving MOUD access for formerly incarcerated individuals by administering injectable naltrexone. However, these services remain underutilized due to several barriers, including lack of awareness of community pharmacist-administered injectable naltrexone services and lack of interagency collaboration among primary care clinics, community pharmacies, and correctional facilities. Pharmacist-led educational meetings with correctional staff and other stakeholders can be an impactful and feasible strategy for addressing these barriers and providing a first step toward better leveraging community pharmacist-administered injectable naltrexone for formerly incarcerated individuals. Utilizing community pharmacist-administered injectable naltrexone can help improve access for formerly incarcerated individuals during community reentry, helping this population avoid negative health outcomes and a cycle of rearrest and reincarceration.

## Data Availability

The raw data supporting the conclusions of this article will be made available by the authors, without undue reservation.
